# Supplementary material for: Language and ethnobiological skills decline precipitously in Papua New Guinea, the world’s most linguistically diverse nation
Source: Proc Natl Acad Sci U S A. 2021 May 26;118(22):e2100096118. doi: 10.1073/pnas.2100096118 (PMC8179190; doi:10.1073/pnas.2100096118)
Supplement: Supplementary File [file pnas.2100096118.sapp.pdf]

PNAS
www.pnas.org

Supplementary Information for

The world's hotspot of linguistic and biocultural diversity under threat

A. Kik*, M. Adamec, A. Y. Aikhenvald, J. Bajzekova, N. Baro, C. Bowern, R. K. Colwell, P. Drozd, P. Duda, S. Ibalim, L. R. Jorge, J. Mogina, B. Ruli, K. Sam, H. Sarvasy, S. Saulei, G. D. Weiblen, J. Zrzavy & V. Novotny*

***Corresponding authors:** Alfred Kik Email: akik@prf.jcu.cz and Vojtech Novotny Email: novotny@entu.cas.cz

This pdf file includes:

SI Materials and Methods

Figures S1 to S6

Tables S1 to S7

Legends for Datasets S1 to S3

SI References

Other supplementary materials for this manuscript include the following:

Datasets S1 (Excel), S2 (Excel), S3 (pdf)

Supplementary Methods and Materials

Language skills and ethnobiological knowledge variables

Methodological details for selected variables:

[A] Language traits: [A1] Language population size: The number of language users was estimated by adjusting the number listed in the *Ethnologue* database (1), interpolated or extrapolated to the year 2000 using annual population growth rates for PNG (2). This standardization was necessary, because the *Ethnologue* estimates date from 1971 – 2019 for individual languages, most often using the PNG National Census data from 2000 (3), and the Summer Institute of Linguistics estimates date from 2003 (Dataset S1). [A2] Language status: We used detailed EGIDS categories as given for each language in *Ethnologue* (1): 1 – EGIDS 3, 2 – EGIDS 4, 3 – EGIDS 5, 4 - EGIDS 6a, 5 – EGIDS 6b, 6 – EGIDS 7 to 10. [A5] Elevation: Each language was characterized by its median elevation (in m, log transformed), obtained from the *Ethnologue* (1) language maps overlaid on geographic maps using Zonal Statistics tool ArcGIS Pro on the Shuttle Radar Topography Mission (SRTM) elevation dataset (4) with spatial resolution 3" latitude x 3" longitude. [A6] Bible translation: We used the lists of languages with at least partial Bible translations (1, 5), but we do not have information on the use of these translations by the surveyed students.

[D] Student traits: [D2-D3] Grade 10 test results from English (D2) and Mathematics (D3): 1 – distinction, 2 – credit, 3 – upper pass, 4 – pass or fail. [D6] The scores were summed within traditional (maximum 10 points) and contemporary technical (maximum 4 points) activities, the totals were rescaled to a 0 – 1 range, and the difference between traditional and contemporary technical skills was used as an explanatory variable. [D7] Best friend's language: We compared the observed values for each surveyed school with the expected proportion of best friends speaking the same language as the informant, calculated as $\sum_{i=1}^{n} p_i^2$, where p_i is the proportion of students speaking language i in the surveyed school. This probability assumes that students choose their friends at school and do so irrespective of the indigenous language they

speak. The overall random expectation of the best friend's language was calculated as the average for all 30 schools surveyed, weighted by their student numbers.

Data verification

Identification of the indigenous language used by each respondent was often difficult. The respondents were often unaware of the name used for their indigenous language by linguists (1) and gave alternative, local names for languages as well as for individual dialects. Further, geographic distribution of many languages in PNG is poorly known, so that language maps remain approximate (1). Villages in PNG also often change their location or name. We therefore integrated information on the language name given by each respondent, the respondent's birthplace, the language and birthplace of the respondent's parents, and the results of the language test naming individual body parts (L1), in order to identify the indigenous language used by the respondent.

We verified the body part test results from 1,990 respondents (32% of the total) speaking the Melpa, Kuman, Enga, and Amele languages (i.e., four of the six languages represented by >100 respondents) with the help of native speakers. The assessment of the responses in other languages was made difficult by the lack of vocabulary lists for many languages (6) and by dialectical differences, which are often poorly documented. Most respondents do not write in their indigenous language, resulting in widely variable spelling in their written responses. The terms for some body parts included in the test may not be widely used in some languages, resulting in particularly high error rates for them. The 47,760 test questions (24 body parts for 1,990 respondents) yielded an answer rate of 81%, including 63% correct answers, 16% answers that referred either to a related body part, or a wider anatomical area (e.g., hand instead of wrist, or toe instead of toenail), and 2% of the answers that were entirely wrong. These data from a few common languages were used to develop a universal protocol applied to all languages. We considered the correct and partly incorrect or vague responses as valid since they reflected some knowledge of the language, as opposed to the entirely incorrect

and non-responses. This strategy was also used for the remaining 388 languages, as far as possible. When we were unsure about the correct term, we accepted the response given by a majority of respondents. In languages represented by a single or a few respondents and lacking linguistic information, we accepted the responses provided as valid, since our detailed analysis of the four common languages indicated that this was predominately the case. We used the same approach to verify indigenous bird names in the tests. The indigenous names of plant species that were freely listed were all accepted as correct, since it was impossible to verify them across 392 languages in our data, most of which are ethnobotanically undocumented (7). Our methods of data verification likely somewhat overestimated language skills and ethnobiological knowledge of the respondents by accepting some erroneous responses as valid. On the other hand, many respondents could be unaccustomed to write in their indigenous language, which could have negatively affected their written test results.

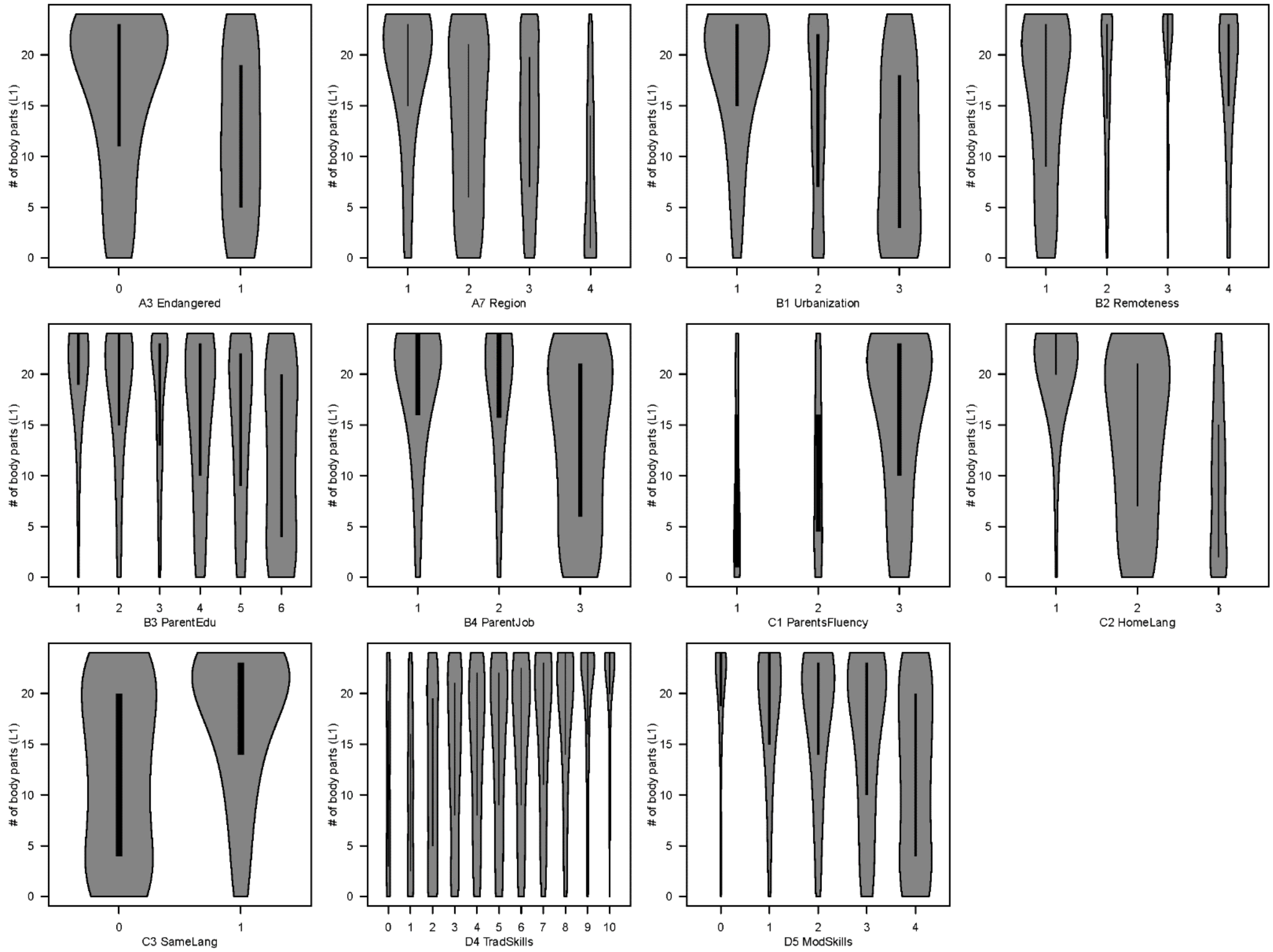

Fig. S1. Effect of the independent variables explaining the indigenous language skills (L1) of students from PNG. Each bar shows the density distribution of the L1 response variable for a given level of the predictor variable, with the width of the bar proportional to the number of students belonging to that class. See Materials and Methods for details on the variables.

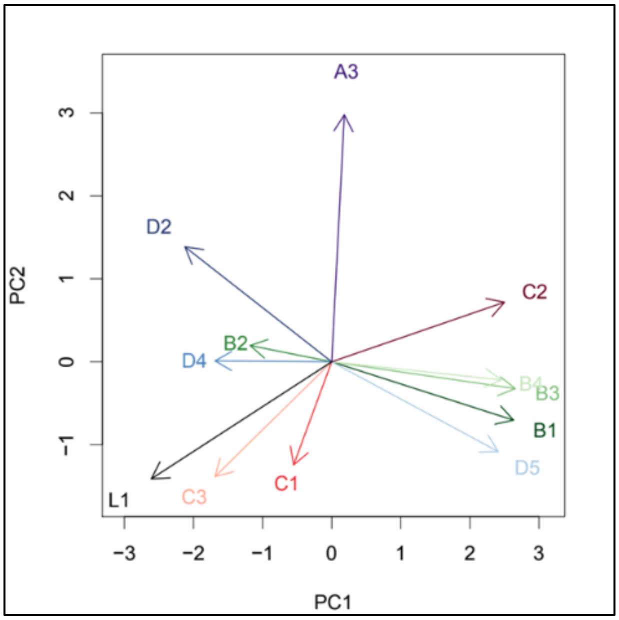

Fig. S2. Correlation of independent variables used to explain language skills. PCA ordination of the 12 independent variables included in the model explaining language skills (L1). All variables were considered numerical, and only linear correlations are visible in this PCA. Variable A4 was excluded as categorical. Arrows represent the direction in which each variable increases along the two PCA axes; their color coding is the same as in Fig. 3. PC1 explained 28.1% and PC2 explained 10.3% of the total variation in the data. See Materials and Methods for the description of variables.

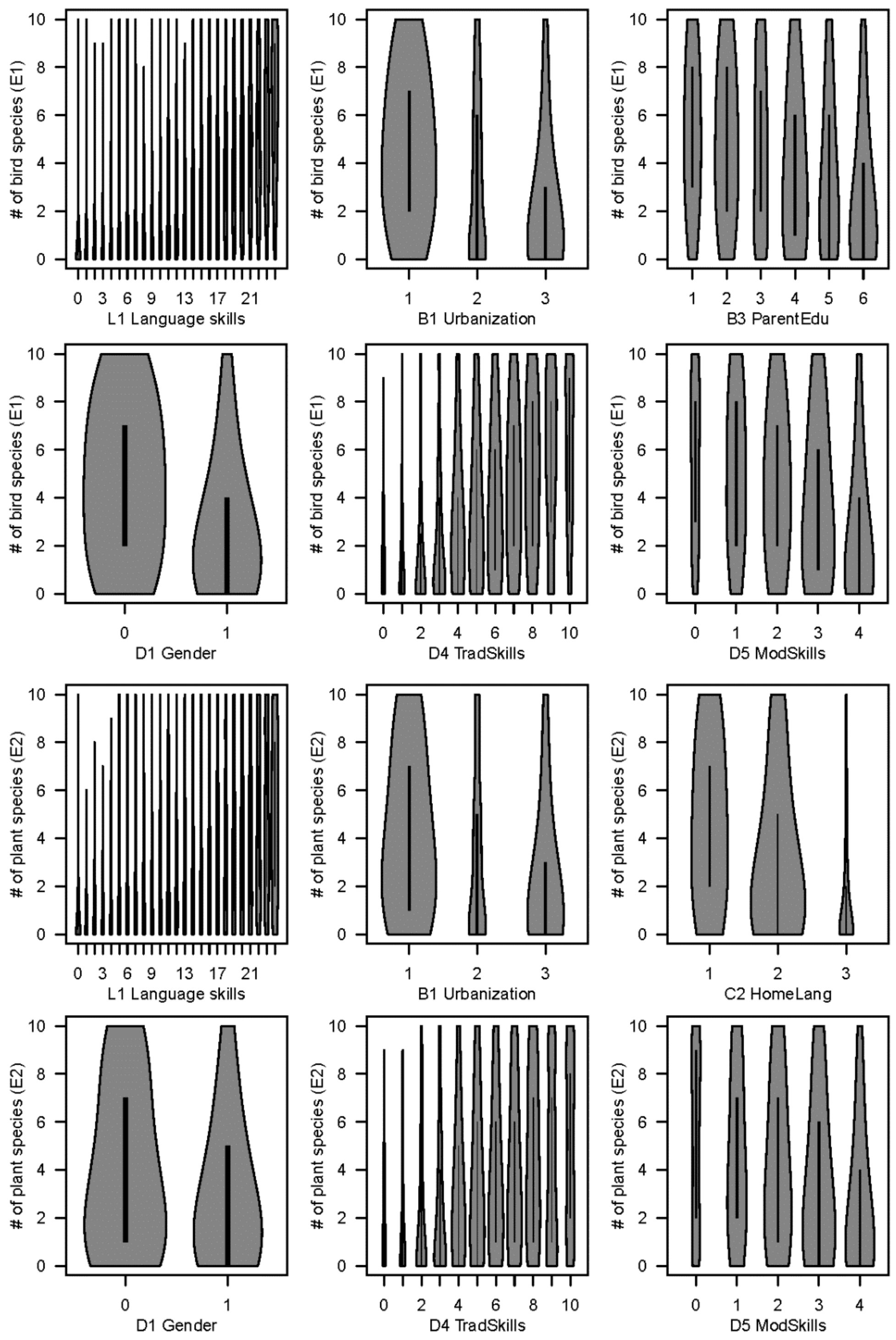

Fig. S3. Effect of the independent variables explaining the ethnobiological knowledge (E1, E2) of students from PNG. Each bar shows the density distribution of the E1 or E2 response variable for a given level of the predictor variable, with the width of the bar proportional to the number of students belonging to that class. See Materials and Methods for details on the variables.

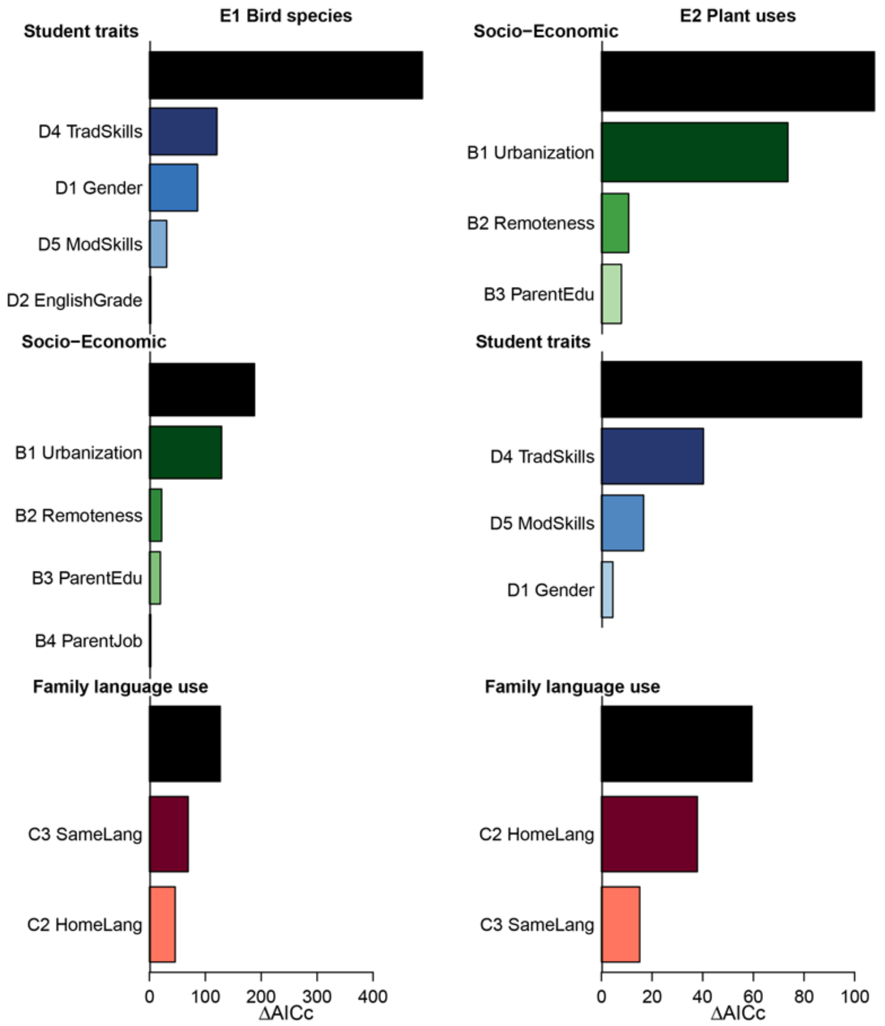

Fig. S4. Effects of language and socio-economic factors on ethnobiological knowledge. Generalized linear mixed models (GLMM) describe variability in the knowledge of bird species (E1, left) and traditional plant uses (E2, right) of students. The models incorporated 11 fixed variables divided into three classes: (B) Socio-economic traits: student's birthplace urbanization (B1) and remoteness (B2), parents' education (B3) and parent's employment (B4); (C) Family language use: parents' language skills (C1), home language use (C2), and whether parents speak the same first language (C3); and (D) Student traits: gender (D1), English skills (D2), traditional skills (hunting, fishing, growing food, house building, medicinal plants) (D4), contemporary technical skills (mobile phone and computer use) (D5). The variables were selected within each class before being included in a global model. The bars show the AIC improvement due to addition of each group (black) and each variable within each group into a model that include all other variables, quantifying the marginal effect of each class/variable. Details on the variables are in Materials and Methods. Only significant variables are shown.

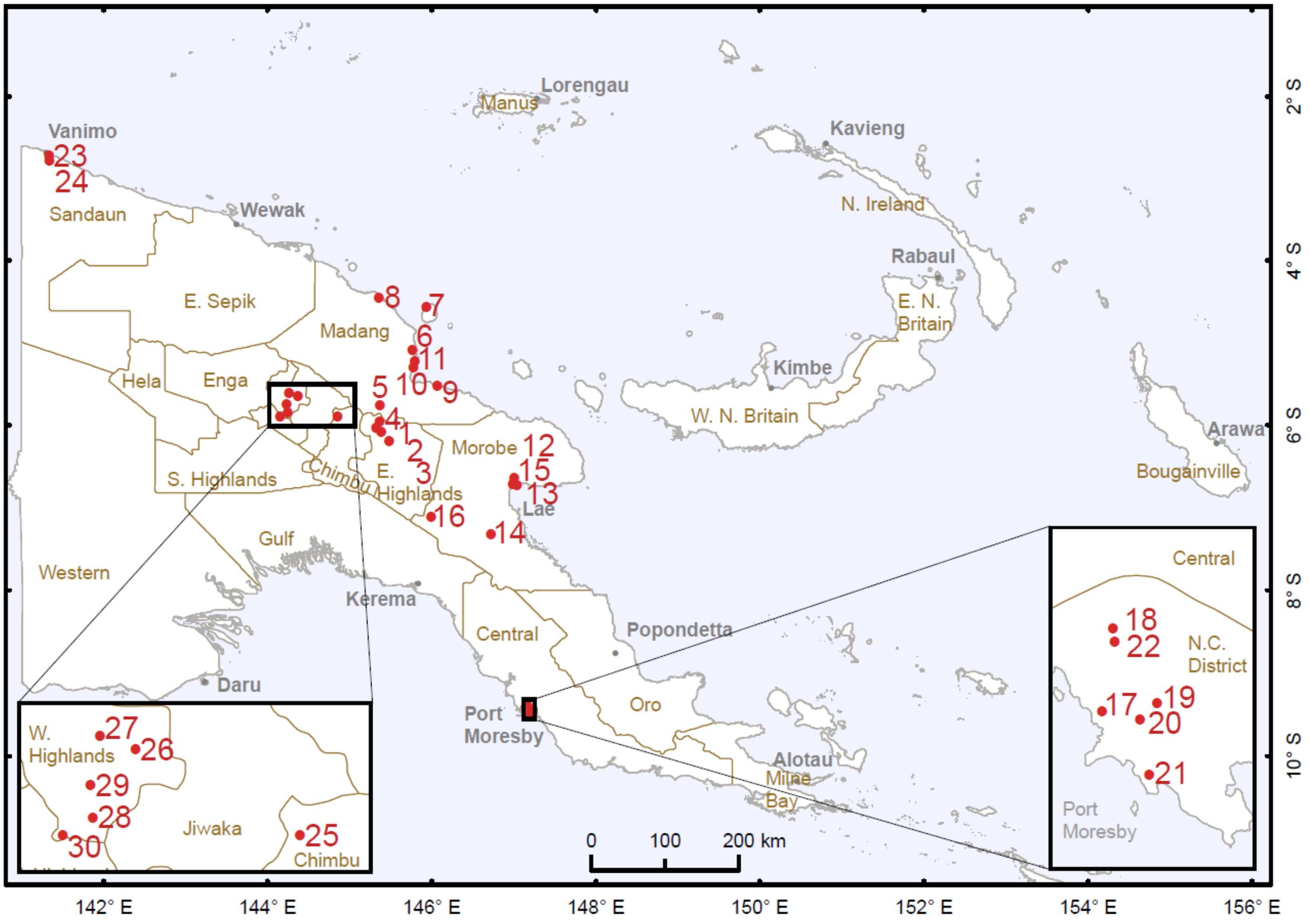

Fig. S5. Location of the 30 secondary schools surveyed in the study. See Table S2 for the details on individual schools.

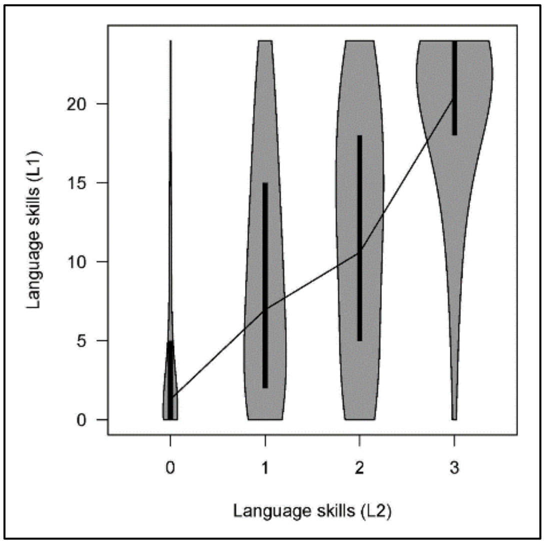

Fig. S6. Correlation between two language skills measures: self-assessed language fluency (L2) and the number of body parts named (L1) for the 6,190 students (Spearman r = 0.58, $P < 0.001$).

Table S1. Socioeconomic and language parameters for respondents. See Materials and Methods for details of variables A1 - D8. Based on the survey of N = 6,190 secondary school students.

Var#	Variable	Value
A1	Language population size [median, Q1-Q3]	3093 [1350, 8500]
A3	Students speaking endangered languages, %	13.9
A6	Students speaking languages with a Bible translation, %	84.0
B1	Students who spent childhood in a village, %	64.7
B2	Student's childhood residence accessible by road %	80.4
B3	Families having ≥1 parent with secondary of higher education, %	63.2
B4	Families with subsistence agriculture income only, %	30.9
C2	Families using indigenous language at home, %	30.0
C3	Families with parents speaking the same indigenous language, %	63.1
D1	Female students, %	41.0
D4	Students with good hunting skills, %	22.4
D4	Students with good fishing skills, %	39.3
D4	Students with good farming skills, %	67.8
D4	Students with good house building skills, %	28.1
D4	Students with good plant medicinal use skills, %	31.0
D5	Students with good mobile phone use skills, %	67.9
D5	Students with good computer use skills, %	28.3
D7	Students speaking the same indigenous language as their best friend	34.8
D8	Students able and wishing to teach indigenous language to their child	87.7
	Student's age [median, Q1-Q3)	19 [18, 20]
	Student's no. of siblings [median, Q1-Q3]	4 [3, 6]
	Families owning land, %	91.7
	Families owning cash crop plantation, %	52.0
	Families owning forest, %	68.5
	Families with access to electricity, %	53.8

Table S2. Secondary schools surveyed in the study. The number of surveyed students, the number of indigenous languages spoken, and the percentage of students speaking the most common language are listed for each school, numbered as in Fig. S5 showing its geographic location.

#	School	Province	Students	Languages	1st lang %
1	Asaroka Lutheran Secondary	Eastern Highlands	149	27	19
2	Benabena Secondary	Eastern Highlands	223	34	39
3	Goroka Secondary School	Eastern Highlands	44	20	23
4	Kabiufa Secondary	Eastern Highlands	137	27	18
5	Braham Secondary	Madang	104	48	13
6	Good Shepherd Lutheran Secondary	Madang	126	51	15
7	Karkar Secondary	Madang	228	48	28
8	Malala Catholic Upper Secondary	Madang	131	46	13
9	Raikos Lutheran Secondary	Madang	100	33	19
10	Transgogol High	Madang	37	18	35
11	Tusbab Secondary	Madang	205	72	12
12	Bumayong Secondary	Morobe	276	84	7
13	Busu Secondary School	Morobe	227	90	8
14	Grace Memorial Secondary	Morobe	212	62	17
15	Lae National High	Morobe	383	124	7
16	Menyamya Secondary	Morobe	182	34	25
17	Badihagwa Secondary Technical	Nat. Capital Distr.	43	28	9
18	Gerehu Secondary	Nat. Capital Distr.	157	52	11
19	Gordons Secondary	Nat. Capital Distr.	157	62	7
20	Jubilee Catholic Secondary	Nat. Capital Distr.	26	20	8
21	Kila Kila Secondary	Nat. Capital Distr.	74	38	12
22	Port Moresby National High	Nat. Capital Distr.	69	33	10
23	Don Bosco Secondary	Sandaun	117	45	24
24	Vanimo Secondary	Sandaun	173	60	21
25	Kerowagi Secondary	Simbu	495	27	76
26	Kitip Secondary	Western Highlands	346	25	82
27	Kwip Dau Secondary	Western Highlands	301	23	79
28	Mount Hagen Secondary	Western Highlands	483	36	66
29	Paglum Adventist Secondary	Western Highlands	189	35	50
30	Togoba Secondary	Western Highlands	422	17	63

Table S3. Model selection results for the candidate models assessing the role of language-related variables A1 – A6, socio-economic variables B1 – B4, family language use variables C1 – C3, and student-related variables D1 – D6 on the language fluency of students. dAICc = delta corrected Akaike Information Criterion, df = degrees of freedom. All models include Student and Language as random factors on the intercept. Note that D7 and D8 were not used in model building.

Variable class	Models – Student trait variables	dAICc	df
Language traits	A1_LogN.2000 + A3_Endangered + A7_RegCode + A8_LogElevMedian	0	9
Language traits	A1_LogN.2000 + A3_Endangered + A6_PNGbible + A7_RegCode + A8_LogElevMedian	0.72	10
Language traits	A1_LogN.2000. + A2_Status + A6_PNGbible + A7_RegCode + A8_LogElevMedian	3.08	13
Language traits	A3_Endangered + A7_RegCode + A8_LogElevMedian	3.27	8
Language traits	A3_Endangered + A6_PNGbible + A7_RegCode + A8_LogElevMedian	5.18	9
Language traits	A1_LogN.2000 + A3_Endangered + A7_RegCode	6.45	8
Language traits	A3_Endangered + A7_RegCode	6.91	7
Language traits	A6_PNGbible + A7_RegCode + A8_LogElevMedian	20.95	8
Language traits	A1_LogN.2000 + A3_Endangered + A8_LogElevMedian	35.78	6
Language traits	Null (random factors + intercept)	72.68	3
Socio-economic	B1_Urbanization + B2_Isolation^2 + B3_ParentEdu + B4_ParentJob^2	0	9
Socio-economic	B1_Urbanization + B2_Isolation^2 + B3_ParentEdu^2 + B4_ParentJob^2	0.96	10
Socio-economic	B1_Urbanization + B2_Isolation^3 + B3_ParentEdu + B4_ParentJob^2	1.82	10
Socio-economic	B1_Urbanization^2 + B2_Isolation^3 + B3_ParentEdu + B4_ParentJob^2	4.80	11
Socio-economic	B1_Urbanization) + B2_Isolation^2 + B3_ParentEdu^2 + B4_ParentJob	12.07	9
Socio-economic	B1_Urbanization + B2_Isolation + B3_ParentEdu + B4_ParentJob^2	24.23	8
Socio-economic	B1_Urbanization + B2_Isolation^2 + B3_ParentEdu	37.19	7
Socio-economic	B1_Urbanization + B3_ParentEdu + B4_ParentJob^2	64.94	7
Socio-economic	B1_Urbanization + B2_Isolation^2 + B4_ParentJob^2	93.49	8
Socio-economic	B2_Isolation^2 + B3_ParentEdu + B4_ParentJob^2	378.61	8
Socio-economic	Null (random factors + intercept)	992.17	3
Language use	C2_HomeLang^2 + C1_ParentMaxFluency + C3_SameLang	0	7
Language use	C2_HomeLang^2 + C1_ParentMaxFluency^2 + C3_SameLang	1.08	8
Language use	C2_HomeLang + C1_ParentMaxFluency + C3_SameLang	32.04	6
Language use	C2_HomeLang + C3_SameLang)	45.72	5
Language use	C2_HomeLang^2 + C1_ParentMaxFluency	249.60	6
Language use	C3_SameLang	533.92	4
Language use	Null (random factors + intercept)	917.26	3
Student traits	D2_GradeEng^2 + D4_TradSkills + D5_ModSkills	0	7
Student traits	D2_GradeEng^3 + D4_TradSkills + D5_ModSkills	0.94	8
Student traits	D1_Gender + D2_GradeEng^3 + D4_TradSkills + D5_ModSkills	2.08	9
Student traits	D2_GradeEng^3 + D3_GradeMath^3 + D4_TradSkills + D5_ModSkills	4.01	11
Student traits	D1_Gender + D2_GradeEng^3 + D3_GradeMath^3 + D4_TradSkills + D5_ModSkills	4.84	12
Student traits	D2_GradeEng^3 + D6_Trad.Mod	12.58	7
Student traits	D2_GradeEng + D4_TradSkills + D5_ModSkills	13.01	6
Student traits	D1_Gender + D2_GradeEng^3 + D3_GradeMath^3 + D6_Trad.Mod	17.00	11
Student traits	D4_TradSkills + D5_ModSkills	48.06	5
Student traits	D1_Gender + D4_TradSkills + D5_ModSkills	49.46	6
Student traits	D1_Gender + D2_GradeEng^3 + D3_GradeMath^3 + D4_TradSkills	199.85	11
Student traits	D1_Gender + D2_GradeEng^3 + D3_GradeMath^3 + D5_ModSkills	246.61	11
Student traits	Null (random factors + intercept)	750.43	3

Table S4. Model selection results for the candidate models assessing the combined role of the four variable classes A – D included in this study on the language fluency of students. dAICc = delta corrected Akaike Information Criterion, df = degrees of freedom. All models include Student and Language as random factors on the intercept.

Combined Model classes	Combined model variables	dAICc	df
All four classes	B1_Urbanization + B2_Isolation^2 + B3_ParentEdu + B4_ParentJob^2 + D2_GradeEng^2 + D4_TradSkills + D5_ModSkills + C2_HomeLang^2 + C1_ParentMaxFluency + C3_SameLang + A1_LogN.2000. + A3_Endangered + A7_RegCode + A8_LogElevMedian	0	23
All four classes significant	B1_Urbanization + B2_Isolation^2 + B3_ParentEdu + B4_ParentJob^2 + D4_TradSkills + D5_ModSkills + C2_HomeLang^2 + C1_ParentMaxFluency + C3_SameLang + A3_Endangered + A7_RegCode	0.14	19
Socio-Economic + Student	B1_Urbanization + B2_Isolation^2 + B3_ParentEdu + B4_ParentJob^2 + D2_GradeEng^2 + D4_TradSkills + D5_ModSkills + C2_HomeLang^2 + C1_ParentMaxFluency + C3_SameLang	48.56	17
Socio-Economic + Family la	B1_Urbanization + B2_Isolation^2 + B3_ParentEdu + B4_ParentJob^2 + D2_GradeEng^2 + D4_TradSkills + D5_ModSkills + A1_LogN.2000. + A3_Endangered + A7_RegCode + A8_LogElevMedian	146.59	19
Socio-Economic + Family la	B1_Urbanization + B2_Isolation^2 + B3_ParentEdu + B4_ParentJob^2 + C2_HomeLang^2 + C1_ParentMaxFluency + C3_SameLang	200.38	13
Socio-Economic + Student	B1_Urbanization + B2_Isolation^2 + B3_ParentEdu + B4_ParentJob^2 + D2_GradeEng^2 + D4_TradSkills + D5_ModSkills	223.06	15
Student traits + Family lang	D2_GradeEng^2 + D4_TradSkills + D5_ModSkills + C2_HomeLang^2 + C1_ParentMaxFluency + C3_SameLang	329.22	11
Socio-Economic + Language	B1_Urbanization + B2_Isolation^2 + B3_ParentEdu + B4_ParentJob^2 + A1_LogN.2000. + A3_Endangered + A7_RegCode + A8_LogElevMedian	586.31	15
Socio-Economic	B1_Urbanization + B2_Isolation^2 + B3_ParentEdu + B4_ParentJob^2	644.16	9
Family language use	C2_HomeLang^2 + C1_ParentMaxFluency + C3_SameLang	719.08	7
Student traits	D2_GradeEng^2 + D4_TradSkills + D5_ModSkills C2_HomeLang^2	885.91	7
Language traits	A1_LogN.2000. + A3_Endangered + A7_RegCode + A8_LogElevMedian	1554.97	16
Null	Null (random factors + intercept)	1636.34	3

Table S5. Variables and their values used in the models extrapolating language fluency. The L1 language skills are extrapolated to the entire 18-20-year-old cohort in PNG (L1A model), to the 18-20-year-old cohort in PNG 30 years in the future (L1B), and to the current average socioeconomic parameters for the Lower-Middle Income countries (L1C). Language fluency (L2 = 3) is extrapolated to the to the entire 18-20-year-old cohort in PNG (L2A model), to the next generation of secondary students in PNG (L2B) and the next 18-20-year-old generation in PNG (L2C). See Materials and Methods for the procedure used to generate values for Adjusted variables.

Model	Var#	Variable	Parameter values	Comments	Ref.	Data
L1A	A3	LangStatus	0 = 0.9782, 1 = 0.0218	Distribution of PNG population among endangered/non-endangered languages based on Language size (A1)	(1)	External
L1A	A4	LangRegion	1 = 0.391, 2 = 0.279, 3 = 0.199, 4 = 0.131	Distribution of PNG population among PNG regions, based on Language size (A1)	(1, 8)	External
L1A	B1	Urbanization	1 = 0.869, 3 = 0.131, 2 = not used	Current PNG urbanization rate	(2)	External
L1A	B2	Remoteness	1 = 0.772, 2 = 0.049, 3 = 0.036, 4 = 0.143	Adjusted by Urbanization (B1)		Adjusted
L1A	B3	ParentEdu	1 = 0.453, 2 = 0.354, 3 = 0.049, 4 = 0.076, 5 = 0.019, 6 = 0.049	Education data for 50-54 years age group in PNG	(9)	External
L1A	B4	ParentJob	1 = 0.57, 2 = 0.125, 3 = 0.303	Job structure in PNG 2009-2010	(10)	External
L1A	C1	ParentLang	0 = 0.001, 1 = 0.009, 2 = 0.014, 3 = 0.976	No change from original data		This study
L1A	C2	HomeLang	1 = 0.469, 2 = 0.521, 3 = 0.010	Adjusted by Urbanization (B1) & Parents' education (B3)		Adjusted
L1A	C3	SameLang	0 = 0.240, 1 = 0.760	Adjusted by Urbanization (B1) & Parents' education (B3)		Adjusted
L1A	D2	EnglishGrade		Not used (part of population does not have Gr 10 test)		Not used
L1A	D4	TraditSkills	0 = 0.010, 1 = 0.011, 2 = 0.041, 3 = 0.069, 4 = 0.106, 5 = 0.123, 6 = 0.154, 7 = 0.144, 8 = 0.162, 9 =0.117, 10 = 0.063	Adjusted by Urbanization (B1) & Parents' education (B3)		Adjusted
L1A	D5	ModernSkills	0 = 0.052, 1 = 0.203, 2 = 0.310, 3 = 0.316, 4 = 0.119	Adjusted by Urbanization (B1) & Parents' education (B3)		Adjusted
L1B	A3	LangStatus	0 = 0.922, 1 = 0.078	Distribution of PNG population among endangered/non-endangered languages based on Language size (A1)	(1)	External
L1B	A4	LangRegion	1 = 0.391, 2 = 0.279, 3 = 0.199, 4 = 0.131	Distribution of PNG population among PNG regions, based on Language size (A1)	(1, 8)	External
L1B	B1	Urbanization	1 = 0.76, 3 = 0.24, 2 = not used	Extrapolation for PNG in 2050	(11)	External
L1B	B2	Remoteness	1 = 0.790, 2 = 0.045, 3 = 0.032, 4 = 0.133	Adjusted by Urbanization (B1)		Adjusted
L1B	B3	ParentEdu	1 = 0.254, 2 = 0.333, 3 = 0.100, 4 = 0.190, 5 = 0.057, 6 = 0.066	Education of 30-34 years old	(9)	External
L1B	B4	ParentJob	1 = 0.57, 2 = 0.125, 3 = 0.303	Data for PNG 2009-2010 were used as no trend was apparent over the past 3[illegible]	(10)	External
L1B	C1	ParentLang	0 = 0.0005, 1 = 0.010, 2 = 0.014, 3 = 0.976	No change from original data		This study
L1B	C2	HomeLang	0 = 0.329, 1 = 0.653, 3 = 0.018	Adjusted by Family language uniformity (C3), Urbanization (B1) and Parents' education (B3)		Adjusted
L1B	C3	SameLang	0 = 0.652, 1 = 0.348	Estimated as the proportion of best friends speaking the same language (D7)		This study
L1B	D2	EnglishGrade		Not used (part of population does not have Gr 10 test)		This study
L1B	D4	TraditSkills	0 = 0.010, 1 = 0.013, 2 = 0.049, 3 = 0.082, 4 = 0.112, 5 = 0.132, 6 = 0.152, 7 = 0.142, 8 = 0.149, 9 =0.104, 10 = 0.055	Adjusted by Urbanization (B1) & Parents' education (B3)		Adjusted
L1B	D5	ModernSkills	0 = 0.038, 1 = 0.167, 2 = 0.287, 3 = 0.343, 4 = 0.165	Adjusted by Urbanization (B1) & Parents' education (B3)		Adjusted
L1C	A3	LangStatus	0 = 0.922, 1 = 0.078	Distribution of PNG population among endangered/non-endangered languages based on Language size (A1)	(1)	External
L1C	A4	LangRegion	1 = 0.391, 2 = 0.279, 3 = 0.199, 4 = 0.131	Distribution of PNG population among PNG regions, based on Language size (A1)	(1, 8)	External
L1C	B1	Urbanization	1 = 0.584, 2 = not used, 3 = 0.416	Lower-middle income countries in 2020	(11)	External
L1C	B2	Remoteness	1 = 0.818, 2 = 0.038, 3 = 0.028, 4 = 0.116	Adjusted by Urbanization (B1)		This study
L1C	B3	ParentEdu	1 = 0.247, 2.5 = 0.430, 4.5 = 0.276, 6 = 0.047	Education attainment in 30-40yrs old population in lower-middle income countries	(12)	External
L1C	B4	ParentJob	1.5 = 0.39, 3 = 0.61	1.5 = work in agriculture, 3 = employment in other sectors	(12)	External
L1C	C1	ParentLang	0 = 0.0005, 1 = 0.010, 2 = 0.014, 3 = 0.976	No change from original data		This study
L1C	C2	HomeLang	0 = 0.269, 1 = 0.702, 3 = 0.029	Adjusted by Family language uniformity (C3), Urbanization (B1) and Parents' education (B3)		Adjusted
L1C	C3	SameLang	0 = 0.652, 1 = 0.348	Estimated as the proportion of best friends speaking the same language (D7)		This study
L1C	D4	TraditSkills	0 = 0.010, 1 = 0.016, 2 = 0.056, 3 = 0.097, 4 = 0.117, 5 = 0.135, 6 = 0.151, 7 = 0.130, 8 = 0.144, 9 =0.093, 10 = 0.051	Adjusted by Urbanization (B1) & Parents' education (B3)		Adjusted
L1C	D5	ModernSkills	0 = 0.049, 1 = 0, 2 = 0.608, 3 = 0.142, 4 = 0.201	Based on access to mobile phone and computer and access to internet	(12)	External

Table S5. Continued

Model	Var#	Variable	Parameter values	Comments	Ref.	Data
L2A	B1	Urbanization	1 = 0.869, 3 = 0.131, 2 = not used	Current urbanization rate	(2)	External
L2A	B3	ParentEdu	1 = 0.453, 2 = 0.354, 3-6 = 0.193	Education data for 50-54 years age group in PNG	(9)	External
L2A	B4	ParentJob	1-2 = 0.695, 3 = 0.305	Job structure in PNG 2009-2010	(10)	External
L2A	C2	HomeLang	1 = 0.467 2-3 = 0.533	Adjusted by Urbanization (B1) & Parents' education (B3)		Adjusted
L2A	C3	SameLang	0 = 0.244, 1 = 0.756	Adjusted by Urbanization (B1) & Parents' education (B3)		Adjusted
L2B	B1	Urbanization	1 = 0.76, 3 = 0.24, 2 = not used	Extrapolation for PNG in 2050	(11)	External
L2B	B3	ParentEdu	1 = 0, 2 = 0, 3 0, 4 = 0, 5 = 0.532, 6 = 0.468	Proportion of secondary school graduates with tertiary education for 25-34 age group in PNG	(9)	External
L2B	C2	HomeLang	1 = 0.306, 2-3 = 0.694	Adjusted by Family language unifornity (C3), Urbanization (B1), Parents' education (B3), and Parent's Language skills (C1) (vernacular language use only for those fluent in it, L2 = 3)		Adjusted
L2B	C3	SameLang	0 = 0.556, 1 = 0.444	The proportion of best friends speaking the same language for those fluent in language (D7)		This study
L2C	B1	Urbanization	1 = 0.76, 3 = 0.24, 2 = not used	Extrapolation for PNG in 2050	(11)	External
L2C	B3	ParentEdu	1 = 0.254, 2 = 0.333, 3 = 0.100, 4 = 0.190, 5 = 0.057, 6 = 0.066	Education of 30-34 years old in PNG	(9)	External
L2C	B4	ParentJob	1 = 0.572, 2 = 0.125, 3 = 0.303	Job structure in PNG 2009-2010 used as no trends in PNG jobs apparent	(11)	External
L2C	C2	HomeLang	1 = 0.388 2-3 = 0.612	Adjusted by Urbanization (B1), Parents' education (B3) and Parent's language skills (C1) (vernacular language use only for those fluent in it, L2 = 3)		Adjusted
L2C	C3	SameLang	0 = 0.297, 1 = 0.703	Adjusted by Urbanization (B1), Parents' education (B3) and Parent's language skills (C1) (vernacular language use only for those fluent in it, L2 = 3)		Adjusted

Table S6. Model selection results for the candidate models assessing the role of socioeconomic variables B1 – B4, family language use variables C1 – C3, and student-related variables D1 – D6 on the knowledge of bird species (E1) and traditional plant use (E2) by students. dAICc = delta corrected Akaike Information Criterion, df = degrees of freedom. All models include Student and Language as random factors on the intercept.

Taxon	Variable class	Models – Student trait variables	dAICc	df
Birds	Socio-economic	B1_Urbanization + B2_Isolation^2 + B3_ParentEdu^2 + B4_ParentJob^2	0	10
Birds	Socio-economic	B1_Urbanization + B2_Isolation^3 + B3_ParentEdu + B4_ParentJob^2	4.60	10
Birds	Socio-economic	B1_Urbanization + B2_Isolation^2 + B3_ParentEdu + B4_ParentJob^2	4.80	9
Birds	Socio-economic	B1_Urbanization^2 + B2_Isolation^3 + B3_ParentEdu + B4_ParentJob^2	4.89	11
Birds	Socio-economic	B1_Urbanization + B2_Isolation^2 + B3_ParentEdu^2 + B4_ParentJob	7.63	9
Birds	Socio-economic	B1_Urbanization + B2_Isolation^2 + B3_ParentEdu	17.15	7
Birds	Socio-economic	B1_Urbanization + B2_Isolation + B3_ParentEdu + B4_ParentJob^2	28.68	8
Birds	Socio-economic	B1_Urbanization + B3_ParentEdu + B4_ParentJob^2	51.05	7
Birds	Socio-economic	B1_Urbanization + B2_Isolation^2 + B4_ParentJob^2	90.28	8
Birds	Socio-economic	B2_Isolation^2 + B3_ParentEdu + B4_ParentJob^2	357.64	8
Birds	Socio-economic	Null (random factors + intercept)	835.38	3
Birds	Language use	C2_HomeLang^2 + C1_ParentMaxFluency^2 + C3_SameLang	0	8
Birds	Language use	C2_HomeLang + C1_ParentMaxFluency + C3_SameLang	2.54	6
Birds	Language use	C2_HomeLang^2 + C1_ParentMaxFluency + C3_SameLang	6.56	7
Birds	Language use	C2_HomeLang + C3_SameLang	8.43	5
Birds	Language use	C2_HomeLang, 2 + C1_ParentMaxFluency, 1	132.04	6
Birds	Language use	C3_SameLang	349.94	4
Birds	Language use	Null (random factors + intercept)	569.16	3
Birds	Student traits	D1_Gender + D2_GradeEng^3 + D4_TradSkills + D5_ModSkills	0	9
Birds	Student traits	D1_Gender + D2_GradeEng^2 + D4_TradSkills + D5_ModSkills	1.99	8
Birds	Student traits	D1_Gender + D2_GradeEng^3 + D3_GradeMath^3 + D4_TradSkills + D5_ModSkills	5.17	12
Birds	Student traits	D1_Gender + D2_GradeEng + D4_TradSkills + D5_ModSkills	19.08	7
Birds	Student traits	D1_Gender + D2_GradeEng^3 + D3_GradeMath^3 + D6_Trad.Mod	22.33	11
Birds	Student traits	D1_Gender + D4_TradSkills + D5_ModSkills	42.13	6
Birds	Student traits	D2_GradeEng^3 + D4_TradSkills + D5_ModSkills	81.35	8
Birds	Student traits	D2_GradeEng^3 + D3_GradeMath^3 + D4_TradSkills + D5_ModSkills	81.96	11
Birds	Student traits	D2_GradeEng^2 + D4_TradSkills + D5_ModSkills	84.53	7
Birds	Student traits	D2_GradeEng + D4_TradSkills + D5_ModSkills	100.34	6
Birds	Student traits	D4_TradSkills + D5_ModSkills	125.79	5
Birds	Student traits	D2_GradeEng^3 + D6_Trad.Mod	162.11	7
Birds	Student traits	D1_Gender + D2_GradeEng^3 + D3_GradeMath^3 + D4_TradSkills	180.34	11
Birds	Student traits	D1_Gender + D2_GradeEng^3 + D3_GradeMath^3 + D5_ModSkills	243.15	11
Birds	Student traits	Null (random factors + intercept)	1066.42	3

Table S6. Continued.

Taxon	Variable class	Models – Student trait variables	dAICc	df
Plants	Socio-economic	B1_Urbanization + B2_Isolation^2 + B3_ParentEdu + B4_ParentJob	0	8
Plants	Socio-economic	B1_Urbanization + B2_Isolation^2 + B3_ParentEdu + B4_ParentJob^2	0.33	9
Plants	Socio-economic	B1_Urbanization + B2_Isolation^2 + B3_ParentEdu^2 + B4_ParentJob	1.49	9
Plants	Socio-economic	B1_Urbanization + B2_Isolation^2 + B3_ParentEdu^2 + B4_ParentJob^2	1.95	10
Plants	Socio-economic	B1_Urbanization + B2_Isolation^3 + B3_ParentEdu + B4_ParentJob^2	2.27	10
Plants	Socio-economic	B1_Urbanization^2 + B2_Isolation^3 + B3_ParentEdu + B4_ParentJob^2	4.27	11
Plants	Socio-economic	B1_Urbanization + B2_Isolation^2 + B3_ParentEdu	5.12	7
Plants	Socio-economic	B1_Urbanization + B2_Isolation + B3_ParentEdu + B4_ParentJob	14.16	7
Plants	Socio-economic	B1_Urbanization + B2_Isolation + B3_ParentEdu + B4_ParentJob^2	14.22	8
Plants	Socio-economic	B1_Urbanization + B3_ParentEdu + B4_ParentJob^2	24.50	7
Plants	Socio-economic	B1_Urbanization + B2_Isolation^2 + B4_ParentJob^2	25.19	8
Plants	Socio-economic	B1_Urbanization + B2_Isolation^2 + B4_ParentJob	27.28	7
Plants	Socio-economic	B2_Isolation^2 + B3_ParentEdu + B4_ParentJob^2	181.05	8
Plants	Socio-economic	Null (random factors + intercept)	392.61	3
Plants	Language use	C2_HomeLang^2 + C1_ParentMaxFluency + C3_SameLang	0	7
Plants	Language use	C2_HomeLang + C3_SameLang	0.57	5
Plants	Language use	C2_HomeLang^2 + C1_ParentMaxFluency^2 + C3_SameLang	1.70	8
Plants	Language use	C2_HomeLang + C1_ParentMaxFluency + C3_SameLang	1.72	6
Plants	Language use	C2_HomeLang^2 + C1_ParentMaxFluency	37.32	6
Plants	Language use	+C3_SameLang	185.97	4
Plants	Language use	Null (random factors + intercept)	263.85	3
Plants	Student traits	D1_Gender + D2_GradeEng^2 + D4_TradSkills + D5_ModSkills	0	8
Plants	Student traits	D1_Gender + D2_GradeEng^3 + D4_TradSkills + D5_ModSkills	0.25	9
Plants	Student traits	D1_Gender + D2_GradeEng^3 + D3_GradeMath^3 + D4_TradSkills + D5_ModSkills	1.79	12
Plants	Student traits	D1_Gender + D2_GradeEng^3 + D3_GradeMath^3 + D6_Trad.Mod	2.63	11
Plants	Student traits	D2_GradeEng^2 + D4_TradSkills + D5_ModSkills	5.28	7
Plants	Student traits	D2_GradeEng^3 + D4_TradSkills + D5_ModSkills	5.31	8
Plants	Student traits	D2_GradeEng^3 + D3_GradeMath^3 + D4_TradSkills + D5_ModSkills	6.66	11
Plants	Student traits	D1_Gender + D2_GradeEng + D4_TradSkills + D5_ModSkills	6.70	7
Plants	Student traits	D1_Gender + D4_TradSkills + D5_ModSkills	11.44	6
Plants	Student traits	D2_GradeEng + D4_TradSkills + D5_ModSkills	11.68	6
Plants	Student traits	D2_GradeEng^3 + D6_Trad.Mod	13.18	7
Plants	Student traits	D4_TradSkills + D5_ModSkills	16.85	5
Plants	Student traits	D1_Gender + D2_GradeEng^3 + D3_GradeMath^3 + D5_ModSkills	94.18	11
Plants	Student traits	D1_Gender + D2_GradeEng^2 + D4_TradSkills	94.81	7
Plants	Student traits	D1_Gender + D2_GradeEng^3 + D3_GradeMath^3 + D4_TradSkills	96.51	11
Plants	Student traits	Null (random factors + intercept)	369.48	3

Table S7. Model selection results for the candidate models assessing the combined role of the four variable classes A – D on the knowledge of bird species (E1) and traditional plant use (E2) by students. dAICc = delta corrected Akaike Information Criterion, df = degrees of freedom. All models include Student and Language as random factors on the intercept.

Taxon	Combined Model classes	Combined model variables	dAICc	df
Birds	Socio-Economic + Student	L1_BodyParts^2 + B1_Urbanization + B3_ParentEdu^2 + D1_Gender + D4_TradSkills + D5_ModSkills	0	11
Birds	Socio-Economic + Student	L1_BodyParts^2 + B1_Urbanization + B2_Isolation^2 + B3_ParentEdu^2 + B4_ParentJob^2 + D1_Gender + D2_GradeEng^2 + D4_TradSkills + D5_ModSkills	3.30	17
Birds	Socio-Economic + Student	L1_BodyParts^2 + B1_Urbanization + B2_Isolation^2 + B3_ParentEdu^2 + B4_ParentJob^2 + D1_Gender + D2_GradeEng^2 + D4_TradSkills + D5_ModSkills + C2_HomeLang^2 + C1_ParentMaxFluency^2 + C3_SameLang	4.10	22
Birds	Student traits + Family lan	L1_BodyParts^2 + D1_Gender + D2_GradeEng^2 + D4_TradSkills + D5_ModSkills + C2_HomeLang^2 + C1_ParentMaxFluency^2 + C3_SameLang	44.70	15
Birds	Socio-Economic + Family la	L1_BodyParts^2 + B1_Urbanization + B2_Isolation^2 + B3_ParentEdu^2 + B4_ParentJob^2 + C2_HomeLang^2 + C1_ParentMaxFluency^2 + C3_SameLang	387.30	17
Birds	Language skills	L1_BodyParts^2	572.70	5
Birds	Socio-Economic + Student	B1_Urbanization + B2_Isolation^2 + B3_ParentEdu^2 + B4_ParentJob^2 + D1_Gender + D2_GradeEng^2 + D4_TradSkills + D5_ModSkills	1467.70	15
Birds	Student traits	D1_Gender + D2_GradeEng^2 + D4_TradSkills + D5_ModSkills	1799.80	9
Birds	Socio-Economic	B1_Urbanization + B2_Isolation^2 + B3_ParentEdu^2 + B4_ParentJob^2	2038.40	9
Birds	Family language use	C2_HomeLang^2 + C1_ParentMaxFluency^2 + C3_SameLang	2299.60	6
Birds	Null	Null (random factors + intercept)	2866.20	3
Plants	Socio-Economic + Student	L1_BodyParts^2 + B1_Urbanization + D1_Gender + D4_TradSkills + D5_ModSkills + C2_HomeLang	0	10
Plants	Socio-Economic + Student	L1_BodyParts^2 + B1_Urbanization + B2_Isolation^2 + B3_ParentEdu + B4_ParentJob + D1_Gender + D2_GradeEng^2 + D4_TradSkills + D5_ModSkills + C2_HomeLang + C3_SameLang	5.20	17
Plants	Socio-Economic + Student	L1_BodyParts^2 + B1_Urbanization + B2_Isolation^2 + B3_ParentEdu + B4_ParentJob + D1_Gender + D2_GradeEng^2 + D4_TradSkills + D5_ModSkills	10.10	15
Plants	Student traits + Family lan	L1_BodyParts^2 + B1_Urbanization + B2_Isolation^2 + B3_ParentEdu + B4_ParentJob + D1_Gender + D2_GradeEng^2 + D4_TradSkills + D5_ModSkills + C2_HomeLang + C3_SameLang	28.00	12
Plants	Socio-Economic + Family la	L1_BodyParts^2 + B1_Urbanization + B2_Isolation^2 + B3_ParentEdu + B4_ParentJob + D1_Gender + D2_GradeEng^2 + D4_TradSkills + D5_ModSkills + C2_HomeLang + C3_SameLang	62.00	12
Plants	Socio-Economic + Student	L1_BodyParts^2 + B1_Urbanization + B2_Isolation^2 + B3_ParentEdu + B4_ParentJob + D1_Gender + D2_GradeEng^2 + D4_TradSkills + D5_ModSkills + C2_HomeLang + C3_SameLang	408.60	15
Plants	Socio-Economic	L1_BodyParts^2 + B1_Urbanization + B2_Isolation^2 + B3_ParentEdu + B4_ParentJob + D1_Gender + D2_GradeEng^2 + D4_TradSkills + D5_ModSkills + C2_HomeLang + C3_SameLang	601.00	9
Plants	Student traits	L1_BodyParts^2 + B1_Urbanization + B2_Isolation^2 + B3_ParentEdu + B4_ParentJob + D1_Gender + D2_GradeEng^2 + D4_TradSkills + D5_ModSkills + C2_HomeLang + C3_SameLang	622.90	9
Plants	Family language use	L1_BodyParts^2 + B1_Urbanization + B2_Isolation^2 + B3_ParentEdu + B4_ParentJob + D1_Gender + D2_GradeEng^2 + D4_TradSkills + D5_ModSkills + C2_HomeLang + C3_SameLang	730.00	6
Plants	Language skills	L1_BodyParts^2 + B1_Urbanization + B2_Isolation^2 + B3_ParentEdu + B4_ParentJob + D1_Gender + D2_GradeEng^2 + D4_TradSkills + D5_ModSkills + C2_HomeLang + C3_SameLang	861.50	4
Plants	Null	Null (random factors + intercept)	992.10	3

Dataset S1 Legend

Population size of PNG languages in year 2000.

Dataset S2 Legend

Data for the independent and dependent variables for individual respondents, used for the GLMM analyses.

Dataset S3 Legend

Questionnaire used for data collection.

SI References

1. D. M. Eberhard, G. F., Simons, C. D. Fennig, Eds., *Ethnologue: Languages of the World* (SIL International, ed. 23, 2020).
2. Worldometers, *Papua New Guinea Population* (March 2020); https://www.worldometers.info/world-population/papua-new-guinea-population/.
3. National Statistical Office (NSO), *Papua New Guinea 2000 Census: Final Figures* (NSO, Port Moresby, 2002).
4. A. Jarvis, H. I. Reuter, A. Nelson, E. Guevara, Hole-filled seamless SRTM data V4. (Int. Ctr. Trop. Agric. CIAT (September 2020); http://srtm.csi.cgiar.org.
5. PNG Bible Translation Association (PNGBTA), *Papua New Guinea Scriptures* (March 2020); https://png.bible/.
6. S. J. Greenhill, *TransNewGuinea.org, a Database of the Languages of New Guinea* March 2020); http://transnewguinea.org/.
7. R. Cámara–Leret, Z. Dennehy, Indigenous knowledge of New Guinea's useful plants: a review, *Econ. Bot.* **73**, 405–415 (2019).
8. H. Hammarström, R. Forkel, M. Haspelmath, S. Bank, Sebastian, *Glottolog 4.2.1*. (Max Planck Inst. Science of Human History, Jena (March 2020); http://glottolog.org.

9. National Statistical Office (NSO), *Papua New Guinea Demographic and Health Survey 2016-18* (NSO, 2019).
10. National Statistical Office (NSO). *2009-2010 Papua New Guinea Household Income and Expenditure Survey*, (NSO, 2011).
11. United Nations, *World Urbanization Prospects: The 2018 Revision*, (United Nations, 2019).
12. World Bank, *Data Catalog: Educational Statistics* (March 2020); https://datacatalog.worldbank.org/dataset/education-statistics.
