# Supplementary material for: Language and ethnobiological skills decline precipitously in Papua New Guinea, the world’s most linguistically diverse nation
Source: Proc Natl Acad Sci U S A. 2021 May 26;118(22):e2100096118. doi: 10.1073/pnas.2100096118 (PMC8179190; doi:10.1073/pnas.2100096118)
Supplement: Supplementary File [file pnas.2100096118.sd03.pdf]

The world’s hotspot of linguistic and biocultural diversity under threat

A. Kik*, M. Adamec, A. Y. Aikhenvald, J. Bajzekova, N. Baro, C. Bowern, R. K. Colwell, P. Drozd, P. Duda, S. Ibalim, L.R. Jorge, J. Mogina, B. Ruli, K. Sam, H. Sarvasy, S. Saulei, G.D.Weiblen, J. Zrzavy, V. Novotny*

***Corresponding authors:** Alfred Kik Email: akik@prf.jcu.cz and Vojtech Novotny Email novotny@entu.cas.cz

Dataset S1. Questionnaire used for data collection.

Welcome to the survey of tokples language skills and ethno-biological knowledge in the Madang Province

Thank you for taking part in the survey

Papua New Guinea has the highest number of languages in the world. However, nobody knows how these languages are passed on the new generation of Papua New Guineans. Please, take a short test and help us find out! This study is a project by **Alfred Kik**, based at the New Guinea Binatang Research Center in Madang and the University of PNG in Port Moresby.

Please circle one or several correct answers to each question, or fill the information needed on dotted line: ....

Example: are you a student? [yes] [no]

Your personal information (all information is anonymous, without your name)

1. I am a **[boy]** **[girl]** (circle correct answer)

2. My year of birth **.............** (make a guess if you are not sure)

4. I was born in the village or town: **...**
District **.......................................** Province **.....................................**

5. I spent most of my pre-school years in the village or town: **................................**
It is (circle one answer):

[my parent's village]

[my mother's village]

[my father's village]

[another village]

[government station]

[provincial town or city]

[overseas]

6. If you spent pre-school years in a village or a government station, did it have:
(circle correct answer)

road access by car: [yes] [no]

airstrip: [yes] [no]

boat access by river/sea: [yes] [no]

access ONLY by walking: [yes] [no]

electricity: [yes] [no]

7. My family is now living in the village or town: **..** It is:

[my parent's village]

[my mother's village]

[my father's village]

[another village]

[government station]

[provincial town or city] [overseas]

8. Does your family own land? **[yes] [no]** If yes, do you own land with:

undisturbed forest - big bush: [yes] [no]

logged forest: [yes] [no]

cash crop plantation (coffee, coconut, cocoa, oil palm): [yes] [no]

food gardens: [yes] [no]

grassland/kunai: [yes] [no]

settlement area: [yes] [no]

9. I have **.......** sisters and **........** brothers
(sharing at least one parent, including adopted ones)

10. My grade 10 results (circle your grade for each subject):

English: [distinction] [credit] [upper pass] [pass] [fail]

Mathematics: [distinction] [credit] [upper pass] [pass] [fail]

Science: [distinction] [credit] [upper pass] [pass] [fail]

Social science: [distinction] [credit] [upper pass] [pass] [fail]

11. Do you know how to:

hunt animals in forest: [well] [a little] [no]

catch fish: [well] [a little] [no]

plant gardens: [well] [a little] [no]

build village house: [well] [a little] [no]

make a mumu: [well] [a little] [no]

use plants to treat fever: [well] [a little] [no]

use mobile phone: [well] [a little] [no]

use computer: [well] [a little] [no]

12. What language do you use most of the time in your home?

[English]

[Tok pisin]

[Tok ples]

13. Is your best friend speaking the same tokples as you?

[same]

[different]

[one of us do not speak any tokples]

[both of us do not speak any tokples]

14. What field have you streamed into?

[Science]

[Social science]

Your mother

1. She was born in the village (her asples): **..**
District **.......................................** Province **..**

2. The name of her tokples (or the name of the village where it is spoken): **..**
or circle: **[do not know]**

3. She speaks tokples (circle one answer):
[very well]
[poorly]
[does not speak it but can understand]
[does not speak or understand it at all]

4. Her highest completed education (circle one answer):
[no school]
[elementary school up to grade ....]
[primary school up to grade ....]
[secondary school up to grade ....]
[university certificate] [university diploma] [BSc] [postgraduate]

5. Her present or past jobs (circle one or several answers):
[caring for family/subsistence farming]
[cash crop farming]
[salaried job (write what job): ....]
[own business (write what business): ....]

Your father

1. He was born in the village (his asples): **..**
District **....................................** Province **..**

2. The name of his tokples (or the name of the village where it is spoken): **..**
or circle: **[do not know]**

3. He speaks tokples (circle one answer):
[very well]
[poorly]
[does not speak it but can understand]
[does not speak or understand it at all]

4. His highest completed education (circle one answer):
[no school]
[elementary school up to grade ....]
[primary school up to grade ....]
[secondary school up to grade ....]
[university certificate] [university diploma] [BSc] [postgraduate]

5. His present or past jobs (circle one or several answers):
[caring for family/subsistence farming]
[cash crop farming]
[salaried job (write what job): ....]
[own business (write what business): ....]

Your language skills:

1. Does your mother and father speak the same tokples? **[yes]** **[no]**

2. I speak my mother's tokples (circle one answer):

[very well]

[poorly]

[do not speak it but can understand]

[do not speak or understand it at all]

3. I speak my my father's tokples (circle one answer):

[very well]

[poorly]

[do not speak it but can understand]

[do not speak or understand it at all]

4. I speak also another tokples: **[yes]** **[no]** If yes, then:
Tokples name or the name of the village where spoken: **...**
I speak it:

[very well]

[poorly]

[do not speak it but can understand]

[do not speak or understand it at all]

5. Will you teach tokples to your children? (circle one or several answers)

[no because I do not speak it myself]

[no because it is not a useful skill for my children]

[no because it belongs to an old culture that is now out of date]

[yes because everybody in my village/town area does it]

[yes because it is a useful skill for my children]

[yes because it is a part of my culture]

Language test

We will show you 24 body parts. See how many of them you can name in tokples!
If you speak more than one tokples, use the tokples language you know best. If you do not speak any tokples, circle **[I do not know]** in all cases.

The name of the tokples language used (or the name of the village where it is spoken):

…………………………………………… or circle: **[do not know]**

Body part No. 1

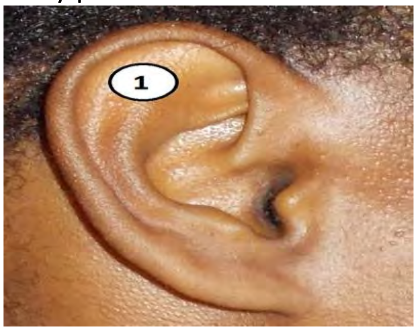

English: Ear

Tokples name: .... or circle: [do not know]

Body part No 2

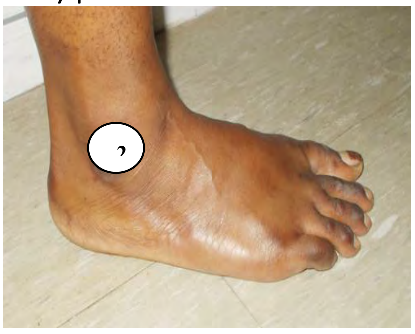

English: Ankle

Tokples name:…………………………………………………………or circle [do not know]

Body part No **3**

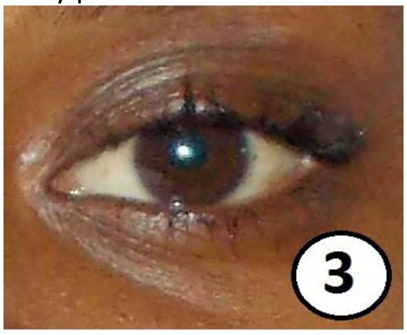

English: Eye

Tokples name:……………………………………………………………or circle [do not know]

Body part No 4

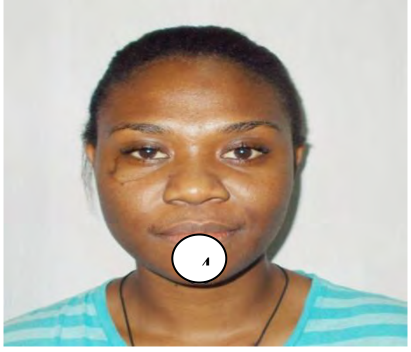

English: Chin

Tokples name:……………………………………………………………or circle [do not know]

Body part No 5

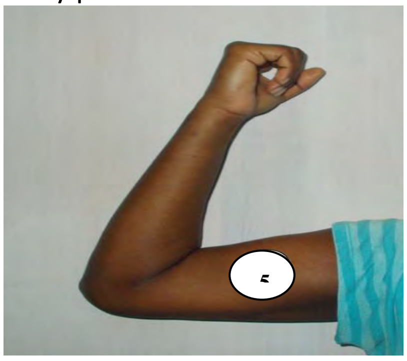

English: Bicep

Tokples name:……………………………………………………………or circle [do not know]

Body part No 6

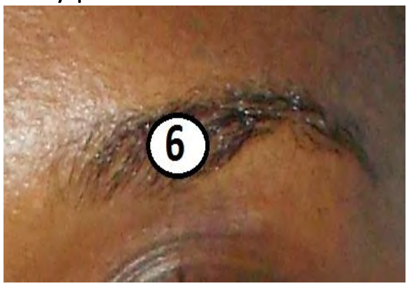

English: Eyebrow

Tokples name:…………………………………………………………….or circle [do not know]

Body part No 7

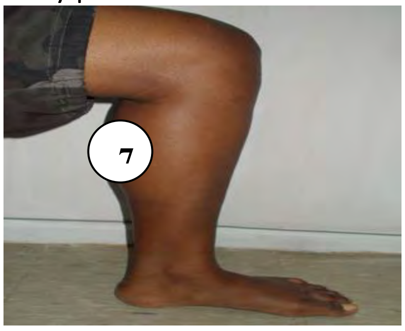

English: Calf

Tokples name:…………………………………………………………….or circle [do not know]

Body part No 8

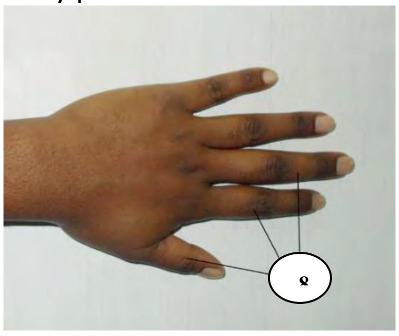
English: Fingers

Tokples name:…………………………………………………………….or circle [do not know]

Body part No 9

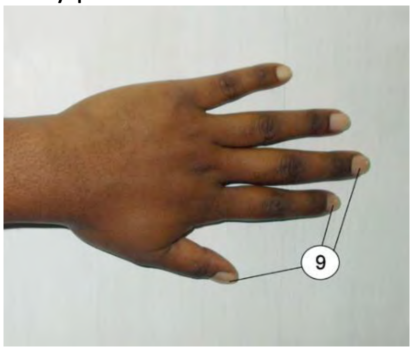

English: Fingernail

Tokples name:…………………………………………………………….or circle [do not know]

Body part No 10

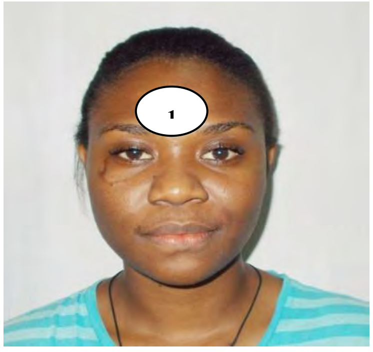
English: Forehead

Tokples name:…………………………………………………………….or circle [do not know]

Body part No 11

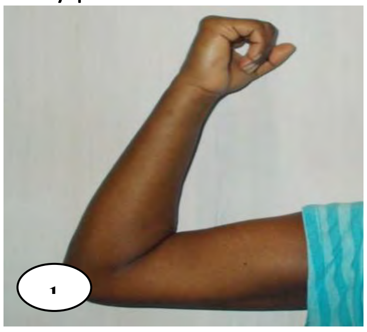

English: Elbow

Tokples name:……………………………………………………………or circle [do not know]

Body part No 12

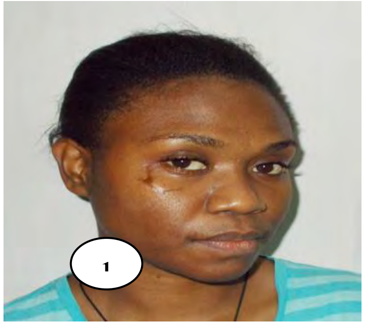

English: Neck

Tokples name:……………………………………………………………or circle [do not know]

Body part No 13

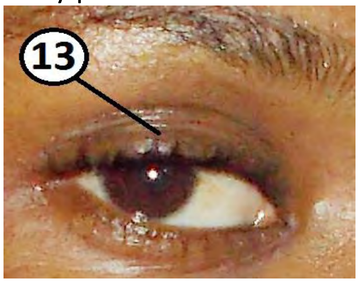

English: Eyelid

Tokples name:……………………………………………………………or circle [do not know]

Body part No 14

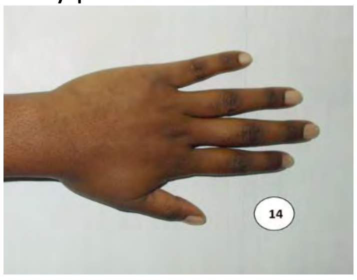

English: Hand

Tokples name:……………………………………………………………or circle [do not know]

Body part No 15

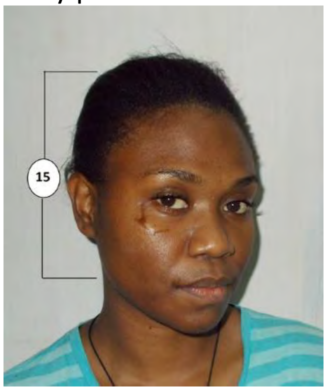

English: Head

Tokples name:……………………………………………………………or circle [do not know]

Body part No 16

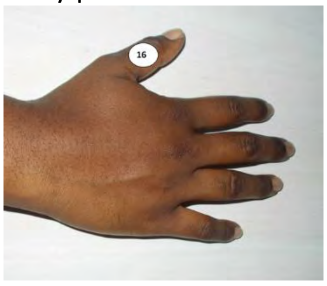

English: Thumb

Tokples name:……………………………………………………………or circle [do not know]

Body part No 17

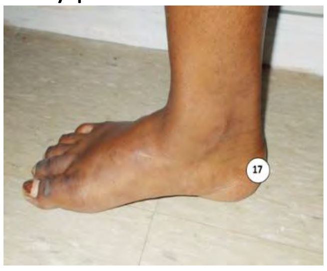

English: Heel

Tokples name:……………………………………………………………or circle [do not know]

Body part No 18

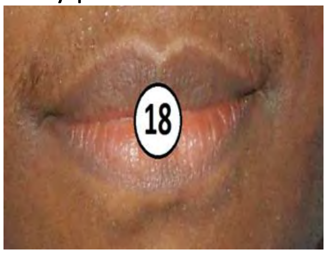

English: Lips

Tokples name:……………………………………………………………or circle [do not know]

Body part No 19

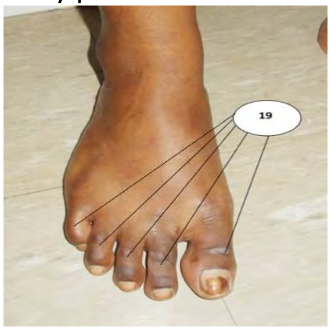

English: Toe

Tokples name:……………………………………………………………or circle [do not know]

Body part No 20

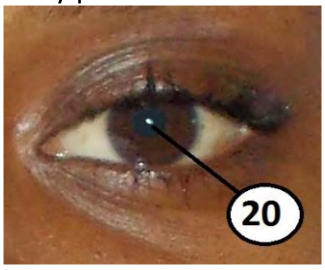

English: Pupil

Tokples name:……………………………………………………………or circle [do not know]

Body part No 21

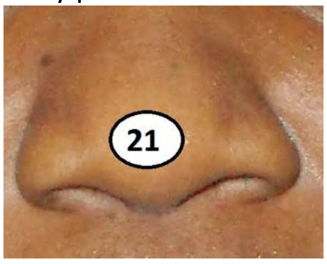

English: Nose

Tokples name:…………………………………………………………….or circle [do not know]

Body part No 22

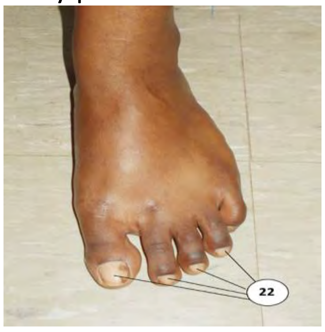

English: Toenails

Tokples name:…………………………………………………………….or circle [do not know]

Body part No 23

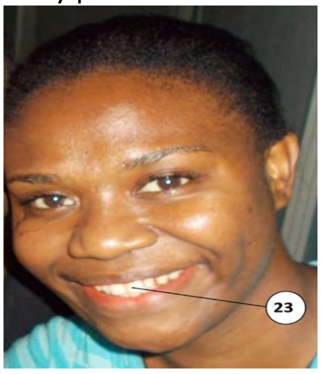

English: Teeth

Tokples name:…………………………………………………………….or circle [do not know]

Body part No 24

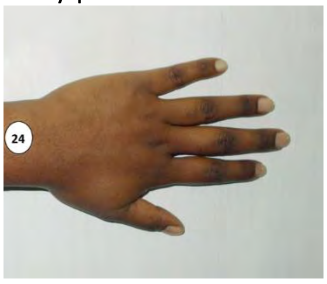

English: Wrist

Tokples name:…………………………………………………………….or circle [do not know]

Birds test

We will show you 20 species of PNG birds from lowlands (nambis), and 20 species from the Highlands. You can choose either **lowland** or **highland** species, depending on which birds you know better. You can also try both groups and we will use the results from the group where you achieved better results.

Please write tokples name for each bird species (or circle [I do not know] option).
Use the same tokples language as you used for the language test.
If you do not know tokples name, try at least Tok Pisin or English

Highlands birds

Bird species No. 1

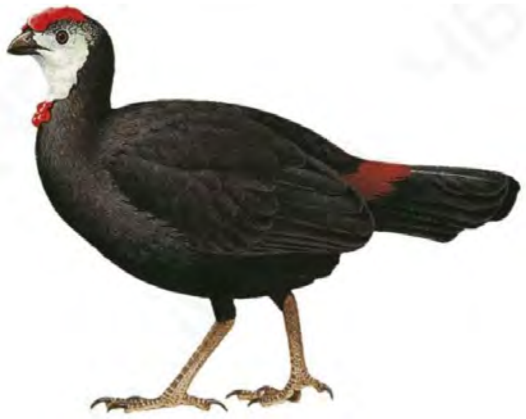

Scientific name: *Aepypodius arfakianus*

Tokples name: .... or circle: [do not know]

Tok Pisin or English name ....

Bird species No. 2

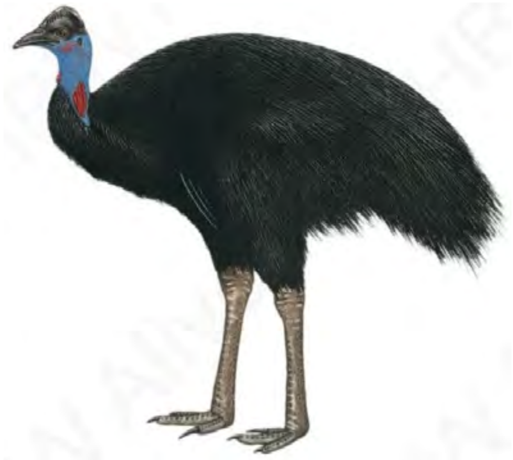

Scientific name: *Casuarius bennetti*

Tokples name: .... or circle: [do not know]

Tok Pisin or English name ....

Bird species No. 3

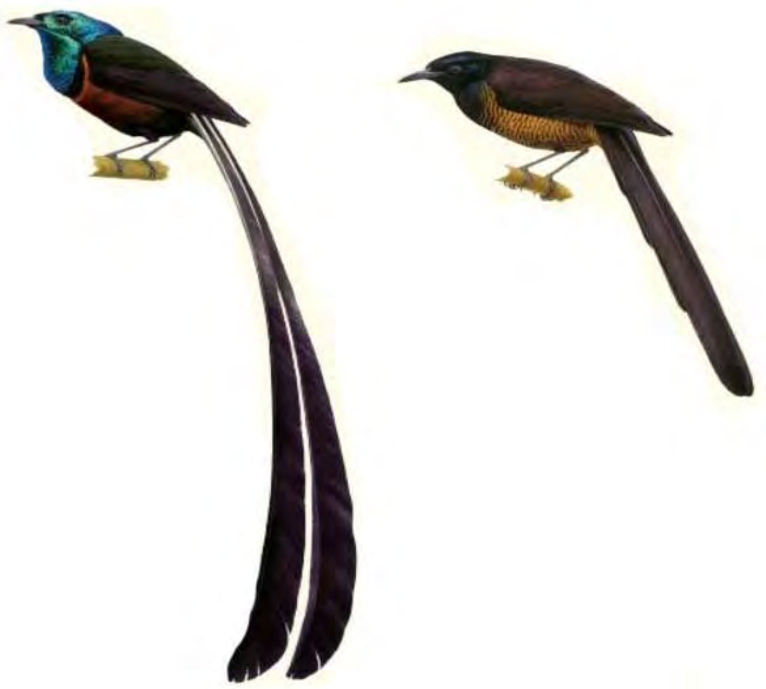

<u>Scientific name:</u> *Astrapia stephaniae*

Tokples name: ………………………………………………… or circle: [do not know]

Tok Pisin or English name …………………………………………………

Bird species No. 4

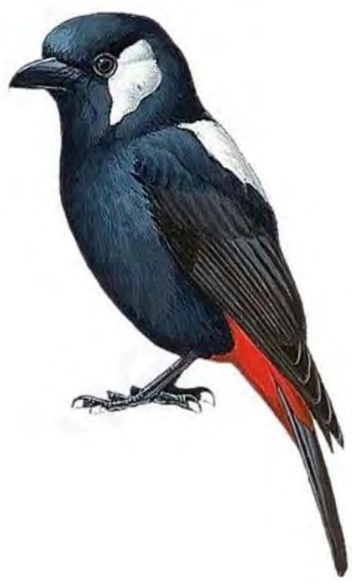

<u>Scientific name:</u> Peltop montanus

Tokples name: ………………………………………………… or circle: [do not know]

Tok Pisin or English name …………………………………………………

Bird species No. 5

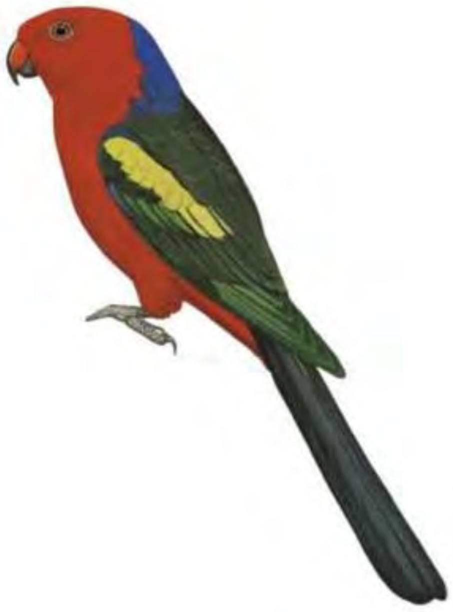
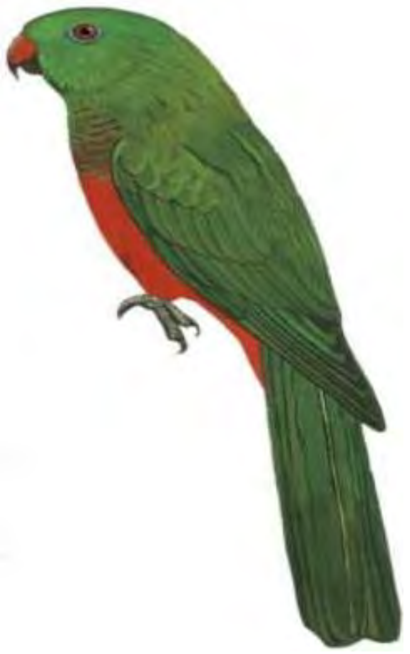

Male Female

Scientific name: Alisterus chloropetrus

Tokples name: .... or circle: [do not know]

Tok Pisin or English name ....

Bird species No. 6

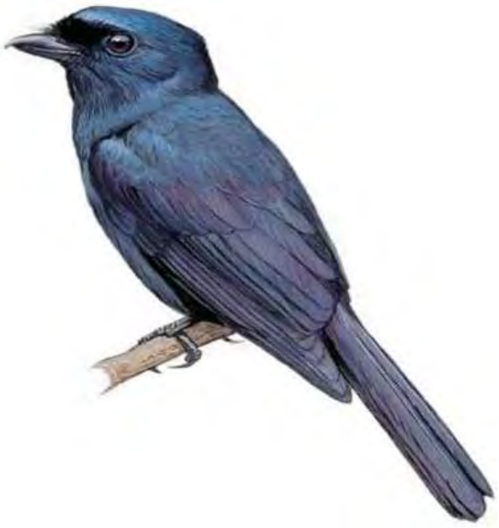

Scientific name: *Chaetorhynchus papuensis*

Tokples name: .... or circle: [do not know]

Tok Pisin or English name ....

Bird species No. 7

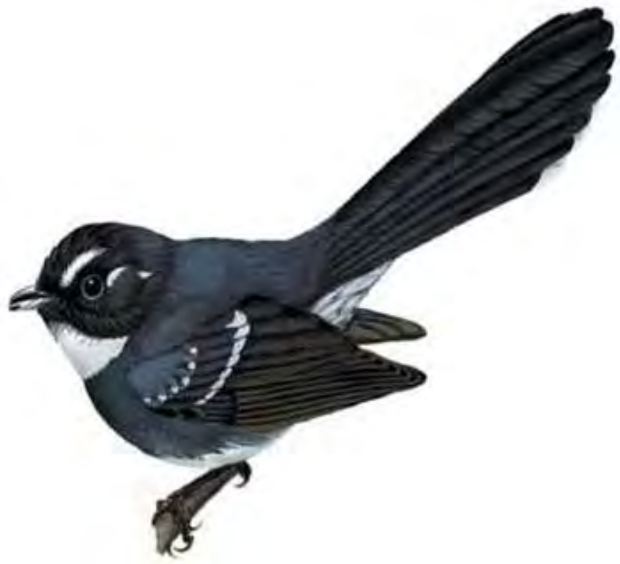

Scientific name: *Rhipidura albolimbata*

Tokples name: ……………………………………………………. or circle: [do not know]

Tok Pisin or English name ………………………………………………….

Bird species No. 8

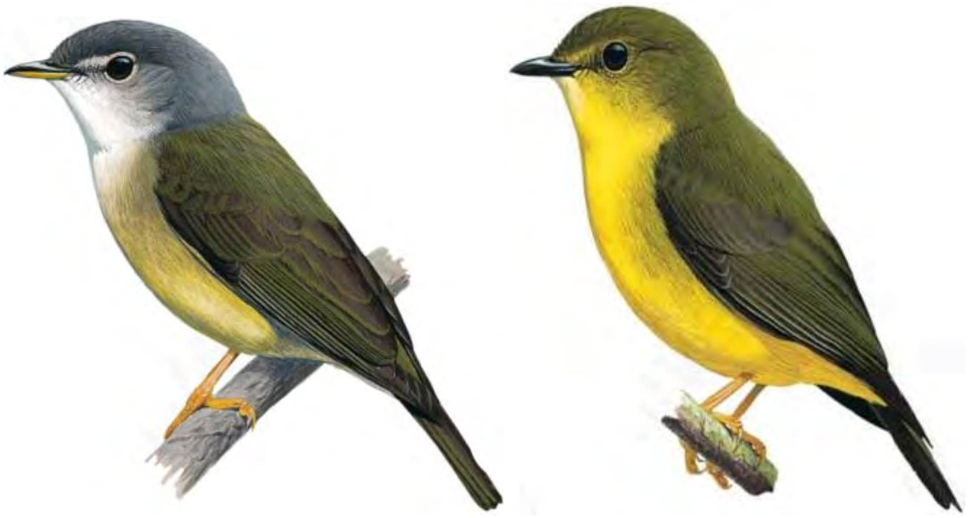

Scientific name: A: *Kempiella griseoceps* B: *Devioeca papuana*

Tokples name for species A: …………………………………………or circle: [do not know]

Tokples name for species B: …………………………………………or circle: [do not know]

Tok Pisin or English name species A ………………………………………………….

Tok Pisin or English name species B………………………………………………….

Bird species No. 9

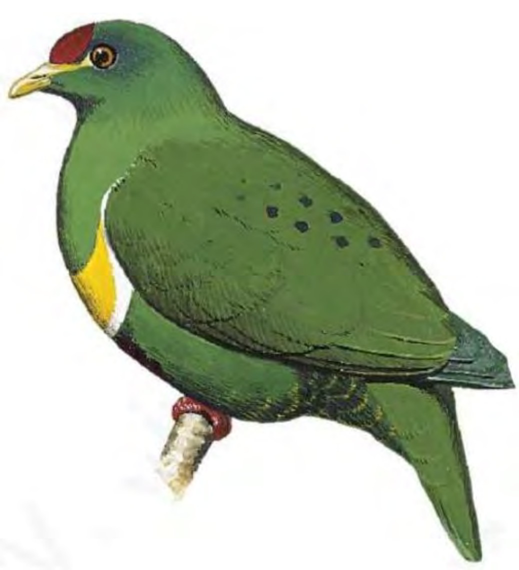

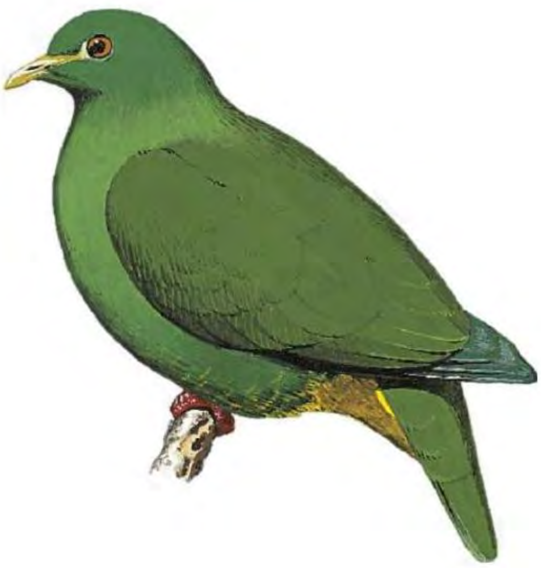

Male Female

Scientific name: *Ptilinopus pulchellus*

Tokples name: .... or circle: [do not know]

Tok Pisin or English name ....

Bird species No. 10

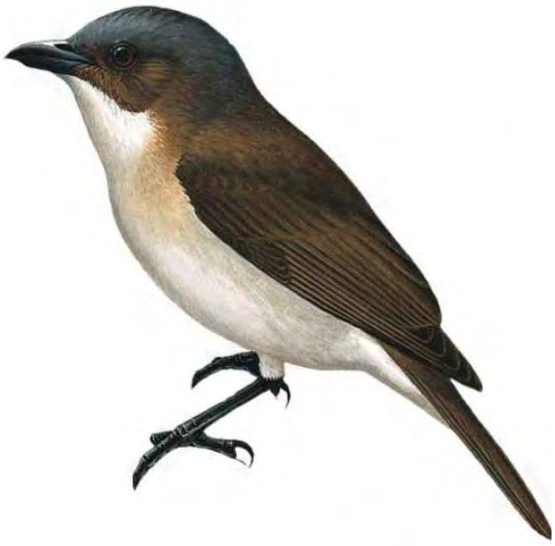

Scientific name: *Pachycephala modesta*

Tokples name: .... or circle: [do not know]

Tok Pisin or English name ....

Lowlands birds:

Bird species No. 1

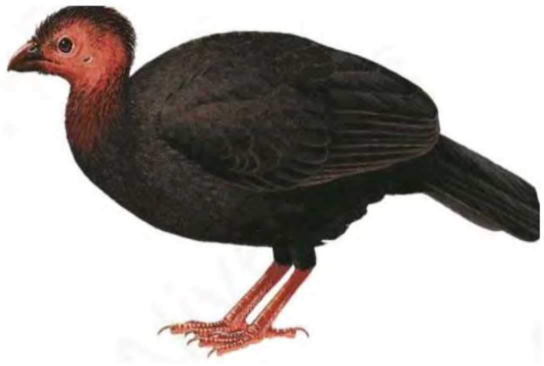

Scientific name: *Talegalla jobiensis*

Tokples name: ……………………………………………………. or circle: [do not know]

Tok Pisin or English name …………………………………………………..

Bird species No. 2

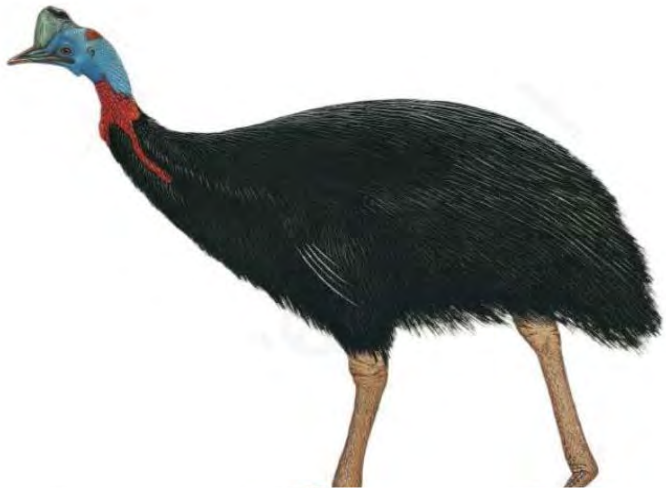

Scientific name: *Casuarius unappendiculatus*

Tokples name: ……………………………………………………. or circle: [do not know]

Tok Pisin or English name …………………………………………………..

Bird species No. 3

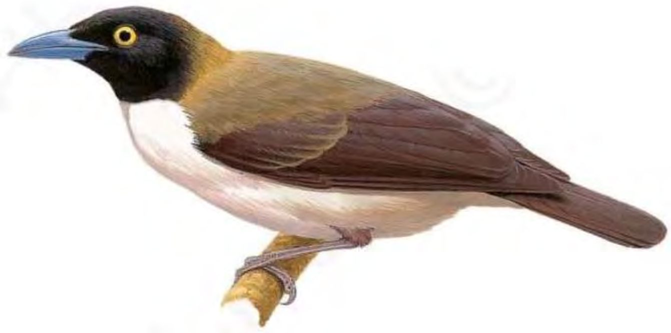

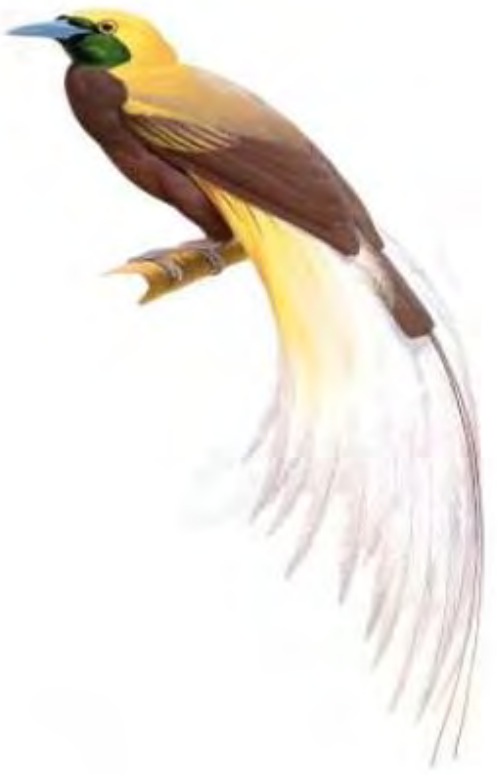

Scientific name: *Paradisaea minor*

Tokples name: .... or cir

Tok Pisin or English name ....

Bird species No. 4

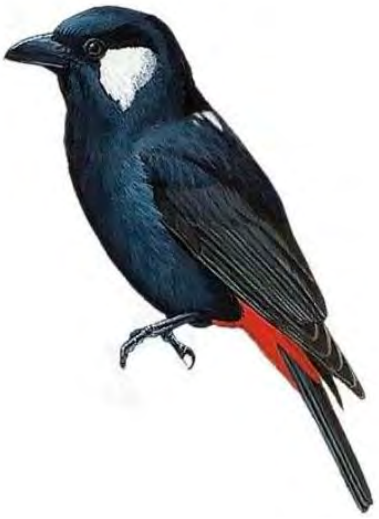

Scientific name: *Peltops blainvillii*

Tokples name: .... or circle: [do not know]

Tok Pisin or English name ....

Bird species No. 5

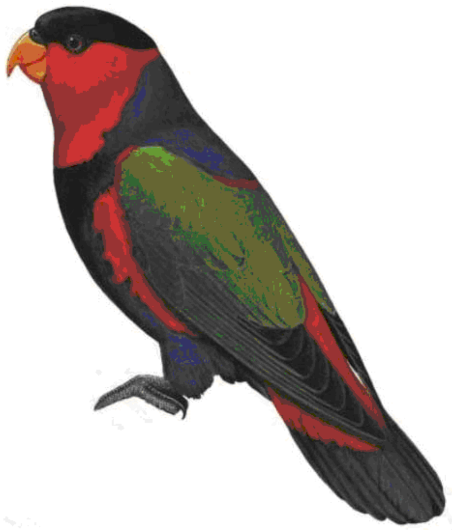

Scientific name: *Lorius lorry*

Tokples name: .... or circle: [do not know]

Tok Pisin or English name ....

Bird species No. 6

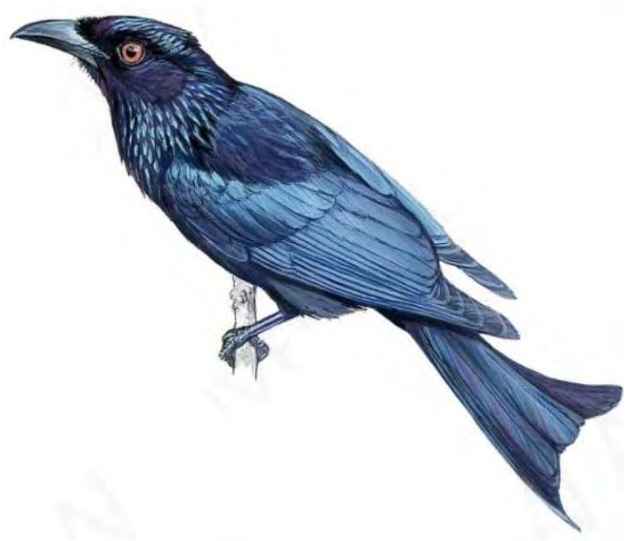

Scientific name: *Dicrurus bracteatus*

Tokples name: .... or circle: [do not know]

Tok Pisin or English name ....

Bird species No. 7

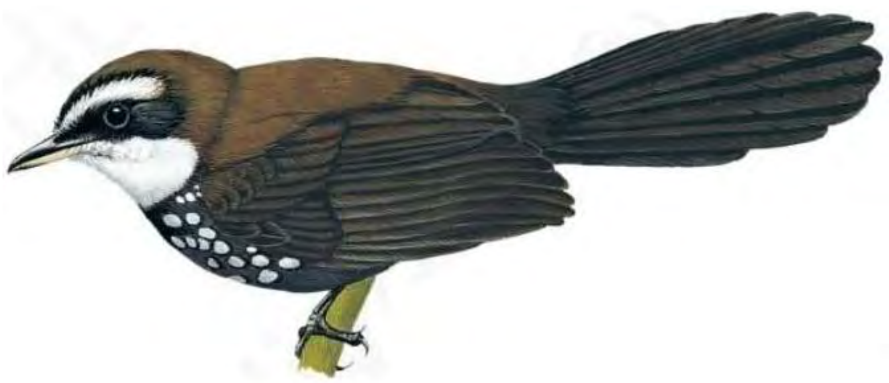

<u>Scientific name:</u> *Rhipidura threnothorax*

Tokples name: .... or circle: [do not know]

Tok Pisin or English name ....

Bird species No. 8

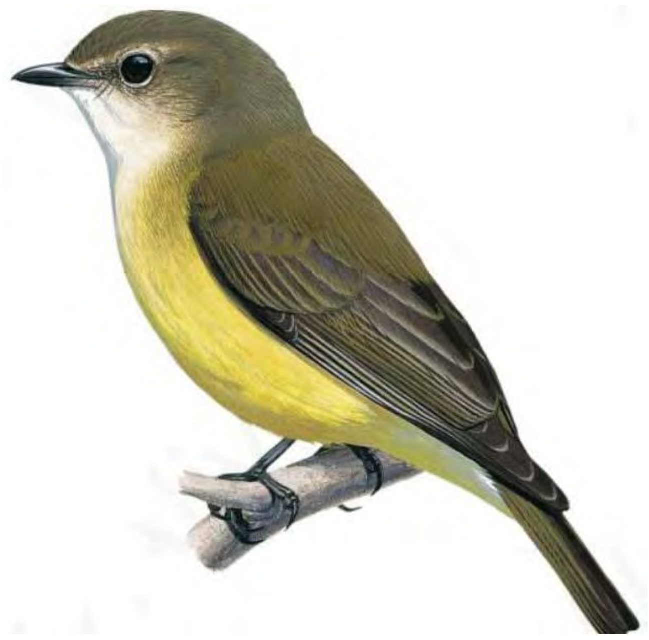

<u>Scientific name:</u> *Microeca flavigaster*

Tokples name: .... or circle: [do not know]

Tok Pisin or English name ....

Bird species No. 9

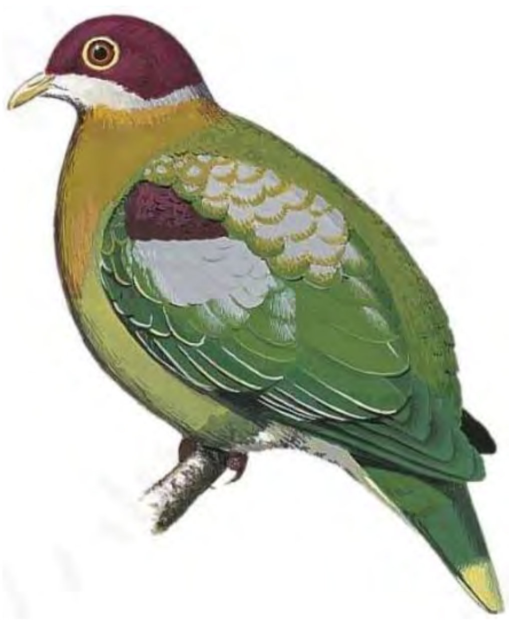

Scientific name: *Ptilinopus ornatus*

Tokples name: ……………………………………………….. or circle: [do not know]

Tok Pisin or English name ………………………………………………..

Bird species No. 10

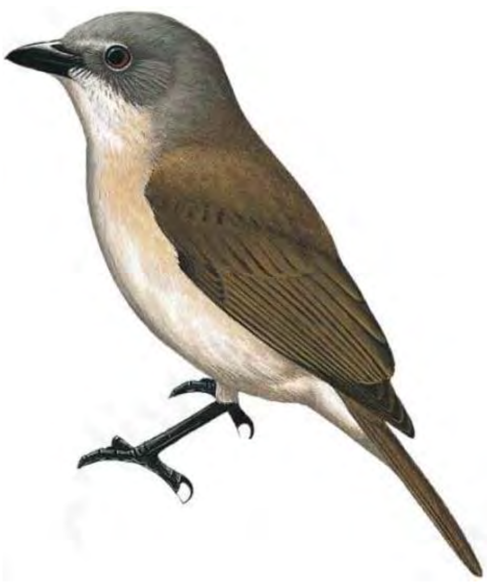

Scientific name: *Pachycephala griseiceps*

Tokples name: ……………………………………………….. or circle: [do not know]

Tok Pisin or English name ………………………………………………..

Plants test

List up to 10 plant species which you know and can use for medicinal, sorcery, or other traditional use. Write the tokples name of each plant (or at least Tok Pisin or English if you do not know tokples name), and describe its use.

	Tokples plant name	Tok Pisin/English name	Plant use (medicinal, sorcery, other traditional, but not food)
1			
2			
3			
4			
5			
6			
7			
8			
9			
10			
